# Identification and validation of a prognostic model based on ferroptosis-associated genes in head and neck squamous cancer

**DOI:** 10.3389/fgene.2022.1065546

**Published:** 2022-12-01

**Authors:** Ming Wei, Yongquan Tian, Yunxia Lv, Guancheng Liu, Gengming Cai

**Affiliations:** ^1^ Department of Otolaryngology Head and Neck Surgery, Xiangya Hospital, Central South University, Changsha, China; ^2^ Department of Thyroid Surgery, The Second Affiliated Hospital to Nanchang University, Nanchang, China; ^3^ Department of Otolaryngology Head and Neck Surgery, Affiliated Hospital of Guilin Medical University, Guilin, China; ^4^ Department of Otolaryngology Head and Neck Surgery, First Affiliated Hospital of Quanzhou, Fujian Medical University, Quanzhou, China

**Keywords:** hNSC, risk score, prognostic model, predicting survival, ferroptosis-associated genes

## Abstract

Ferroptosis is that under the action of ferrous iron or ester oxygenase, unsaturated fatty acids highly expressed on the cell membrane are catalyzed to undergo lipid peroxidation, thereby inducing cell death. In this study, we used ferroptosis marker genes to identify 3 stable molecular subtypes (C1, C2, C3) with distinct prognostic, mutational, and immune signatures by consensus clustering; TP53, CDKN2A, etc. Have higher mutation frequencies in the three subtypes. C3 has a better prognosis, while the C1 subtype has a worse prognosis. WGCNA is used to identify molecular subtype-related gene modules.After filting, we obtained a total of 540 genes related to the module feature vector (correlation>0.7).We performed univariate COX regression analysis on these genes, and identified a total of 97 genes (*p* < 0.05) that had a greater impact on prognosis, including 8 ‘‘Risk” and 89 ‘‘Protective” genes. After using lasso regression, we identified 8 genes (ZNF566, ZNF541, TMEM150C, PPAN, PGLYRP4, ENDOU, RPL23 and MALSU1) as ferroptosis-related genes affecting prognosis. The ferroptosis prognosis-related risk score (FPRS) was calculated for each sample in TCGA-HNSC dataset. The results showed that FPRS was negatively correlated with prognosis.The activated pathways in the PFRS-high group mainly include immune-related pathways and invasion-related pathways. We assessed the extent of immune cell infiltration in patients in our TCGA-HNSC cohort by using the expression levels of gene markers in immune cells. The FPRS-high group had a higher level of immune cell infiltration. We found that the expression of immune checkpoints was significantly up-regulated in the FPRS-low group and the FPRS-high group had a higher probability of immune escape and a lower probability of benefiting from immunotherapy. In this work, we constructed a scoring Ferroptosis-related prognostic model that can well reflect risk and positive factors for prognosis in patients with head and neck squamous cell carcinoma. It can be used to guide individualized adjuvant therapy and chemotherapy for patients with head and neck cancer. Therefore, it has a good survival prediction ability and provides an important reference for clinical treatment.

## 1 Introduction

There are about 600,000 new cases of head and neck malignant tumors every year, and more than 60% of these cases are insidious (Chen et al., 2016; Miller et al., 2016). Although great progress has been made in multidisciplinary treatment in head and neck malignant tumors, 5-year survival rate has not improved significantly, only 40%–50% (Siegel et al., 2011). It is an important means to further understand the molecular mechanism of head and neck tumors and explore new molecular targets such as early diagnosis, prognosis evaluation, and targeted therapy.

Ferroptosis is an iron-dependent, different from apoptosis, necrosis, cell autophagy, a novel form of programmed cell death (Dixon et al., 2014; Stockwell, 2022). The main mechanism of ferroptosis is that under the action of ferrous iron or ester oxygenase, unsaturated fatty acids highly expressed on the cell membrane are catalysed to undergo lipid peroxidation, thereby inducing cell death (Stockwell, 2022). Induction of ferroptosis has received increasing attention as a potential tumor treatment option (Lin et al., 2020). Recent studies have found that many tumor suppressors exert some of their tumor suppressor functions by inducing ferroptosis (Jiang et al., 2015; Chu et al., 2019). p53 inhibits the expression of solute carrier family 7 member 11 (SLC7A11) (Jiang et al., 2015). p53 also causes ALOX12-dependent cell death that is inhibited by ferrostatin-1 and involves the expression of the ferroptosis marker gene Ptgs2 (Chu et al., 2019).

It also reported that the loss of ferroptosis can drive tumorigenesis (Wu et al., 2021). Wang et al. found that CD8 + T cells could drive ferroptosis (Wang et al., 2019). Therefore, activating CD8^+^ T through immune checkpoint blockade therapy to drive ferroptosis to selectively kill tumor cell are obviously beneficial to the improvement of prognosis (Sanmamed and Chen, 2018). Epithelial-mesenchymal transition (EMT) plays an important role in invasion and metastasis, with adverse effects on prognosis. Recent studies have shown that ferroptosis inducers are associated with mesenchymal or metastatic properties of cancer cells and that inhibition of E-cadherin or induction of EMT may contribute to enhanced ferroptosis (Hangauer et al., 2017; Viswanathan et al., 2017; Wu et al., 2019). Inhibition of ferroptosis is an important mechanism of drug resistance in tumor therapy. Inhibition of GPX4 is a well-known method of inducing ferroptosis. Traditional chemotherapeutic drugs inhibit ferroptosis by upregulating GPX4 and X_c-_ system, leading to chemoresistance. However, when some classic chemotherapy drugs are used in combination with ferroptosis inducers, the anticancer effect will be enhanced (Yu et al., 2015). Therefore, ferroptosis can be regarded as an important factor affecting prognosis.

In this study, we used ferroptosis marker genes to identify stable molecular subgroups by consensus clustering type, and further compare the pathway characteristics and immune characteristics between the subtypes. We identified genes associated with the ferroptosis prognostic score by WGCNA and lasso, and further, we constructed a clinical prognostic model of ferroptosis-related prognostic risk score (FPRS). To further improve the prognostic model and survival prediction, we adopted a decision tree model to combine FPRS with clinicopathological features to construct a nomogram for risk assessment of head and neck cancer patients.

## 2 Materials and methods

### 2.1 Date set

We downloaded the HNSC RNA-seq data from the Cancer Genome Atlas public data portal, which finally included a total of 499 primary tumor samples after filtering. The expression data of GSE65858 and GSE42743 were obtained from the Gene Expression Omnibus database. After filtering, 253 and 104 head and neck cancer samples, respectively, were included in the analysis. In this study, we used TCGA-HNSC data as the training set and GSE65858 and GSE42743 datasets as independent validation sets. At the same time, we also downloaded a group of head and neck cancer immunotherapy data GSE78220 as risk mode of immunotherapy response prediction. Here, the ferroptosis-related genes are derived from the FerrDb database, with a total of 259 genes.

### 2.2 Data preprocessing

Perform the following steps to preprocess the RNA-seq data of TCGA: 1)Remove the samples without clinical follow-up information; 2) Remove the samples without survival time; 3) Remove the samples without Status; 4) Convert Ensembl to Gene symbol; 5) Take the median value for expressions with multiple Gene Symbols; Do the following steps to preprocess the GEO data: For the GEO dataset, we downloaded the annotation information of the corresponding chip platform, mapped probes to genes according to the annotation information, and removed probes that matched one probe to multiple genes. When multiple probes matched a gene, the median was taken as the gene expression value. Various datasets and samples showed in such as attachments * .exp.txt, * .cli.txt.

### 2.3 Molecular typing of ferroptosis-related genes

Consensus clustering (ConsensusClusterPlus) was used to construct a consistency matrix and cluster the samples (PMID: 20427518). Using the expression data of ferroptosis -related genes, the molecular subtypes of the samples were obtained. We utilized the pam algorithm and “1-Pearson correlation” as the metric distance and performed 500 bootstraps, each bootstraps process including 80% of the training set patients. The number of clusters was set from 2 to 10, and the optimal classification was determined by calculating the consistency matrix and the consistency cumulative distribution function to obtain the molecular subtypes of the samples.

### 2.4 Construction of weighted gene Co-expression network

Gene co-expression networks were constructed using weighted gene co-expression network analysis (WGCNA) (PMID: 19114008). First, to construct the gene expression similarity matrix, we calculated the absolute value of Pearson’s correlation coefficient between genes i and j using the equation:

$$S_{ij}=\left|\left(1+cor\left(x_{i}+y_{i}\right)\right)\right|/2$$

where i and j represent the expression of genes i and j, respectively. Further, the gene expression similarity matrix was transformed into an adjacency matrix. β is a soft-thresholding parameter and represents Pearson’s correlation coefficient b for each pair of genes [PMID: 17090670]. This step strengthens the strong correlation and weakens the weak correlation from the index level

$$a_{ij}={\left|\left(1+cor\left(x_{i}+y_{i}\right)\right)/2\right|}^{\beta }$$

The representative genes in each module are called characteristic vector genes, referred to as ME, which represent the overall level of gene expression within the module

$$ME=princomp\left(x_{ij}^{q}\right)$$

where i represents the gene in modulus q, and j represents the microarray sample in modulus q. We used Pearson’s correlation for the expression profiles of the genes in all samples, and the ME expression profiles of the signature vector genes to gauge the identity of that gene in the module. We called this module membership (MM)

$${MM}_{i}^{q}=cor\left(x_{i},{ME}^{q}\right)$$

where ME represents the expression profile of gene i.

### 2.5 Construction of the FPRS scoring system to evaluate head and neck cancer samples

(1) Molecular subtype -related modules, where we performed WGCNA analysis using the entire expression profile of TCGA-HNSC, we identified the most relevant modules for molecular subtypes as key modules;
(2) Further, we extracted the genes in the key modules, and selected the genes whose correlation with the module feature vector was greater than 0.7 and had a significant prognosis as the genes related to the ferroptosis phenotype;
(3) The number of genes was further reduced by the method of lasso regression, and the genes related to the prognosis of ferroptosis were obtained;
(4) FPRS scoring system construction. We calculated the FPRS score of each patient using the following formula: FPRS = Σβ i ×Exp i), where i refers to the gene expression level of the ferroptosis prognosis-related gene signature, and β is the Cox regression coefficient of the corresponding gene. According to the best cut-off value of FPRS obtained by the R package survminer, the patients were divided into high and low risk groups of FPRS, the survival curve was drawn by the Kaplan-Meier method for prognostic analysis, and the log-rank test was used to determine the significance of the difference.

### 2.6 Prediction of responsiveness to immunotherapy

We used the TIDE algorithm to verify the effect of IMS on the prediction of clinical responsiveness to ICIs. The TIDE algorithm is a computational method for predicting ICB responsiveness using gene expression profiling [PMID: 30127393]. The TIDE algorithm evaluated three cell types that limit T cell infiltration in tumors, including the M2 subtype of tumor-associated fibroblasts (TAF), myeloid-derived suppressor cells (MDSCs), and tumor-associated macrophages (TAM), and two different mechanisms of tumor immune escape, including the dysfunction score of tumor-infiltrating cytotoxic T lymphocytes (CTLs) (dysfunction) and the rejection score of CTLs by immunosuppressive factors (exclusion).

### 2.7 Gene set enrichment analysis

To study the pathways of different biological processes in different molecular subtypes, we used GSEA for pathway analysis, here we used all candidate gene sets in the Hallmark database [PMID: 26771021] for GSEA.

### 2.8 Calculation of invasive abundance of TME cells

We used the CIBERSORT algorithm (https://cibersort.stanford.edu/) to quantify the relative abundance of 22 immune cells in head and neck cancer. At the same time, we also used ESTIMATE software to calculate the proportion of immune cells.

### 2.9 Real-time PCR

We used TRIzol to obtain total RNA from fresh human head and neck squamous cell carcinoma tissues and paracancerous tissues, and then reverse transcribed into cDNA. The human tissues got from the patients consented to this study during the time of surgery from January 2020 to December 2020 in Quanzhou First Hospital Affiliated to Fujian Medical University. It is considered by the Ethic Committee of the Quanzhou First Hospital Affiliated to Fujian Medical University. Ethics Committee agrees the program to carry out as planned [No. (2019) 109]. The Quantitative real-time PCR was performed on an ABI 7900 system (Takara, Dalian, China) with SYBR Green using SYBR Green RT-PCR Assay (Takara, Dalian, China) and normalized to GAPDH. The following primers were used for PCR: ZNF566, forward primer: 5′-ctc​gac​atc​aca​gaa​ttc​aca​c-3′; and reverse primer: 5′-tct​gat​gtc​gag​tga​agt​ttg​a-3′; TMEM150C, forward primer, 5′-gag​acc​agc​ctg​acc​aat​gtg​aag-3′ and reverse primer, 5′-ctg​cct​ccg​cct​cct​gag​tag-3′; ENDOU, forward primer, 5′-tta​cag​tca​cat​ctc​gcc​ttt​a-3′ and reverse primer, 5′-gga​gta​gag​tgc​aaa​ctc​aaa​c-3′. MALSU1, forward primer, 5′-ttctacccgacacttacatgccatgand-3′ and reverse primer, 5′-cca​cgc​aca​gcc​agt​cat​cag-3′.

### 2.10 Statistical analysis

Statistical analysis was performed using GraphPad Prism 5 and R software (version 3.6.3). Data in the figures are shown as mean ± SD. To compare the expression of tissues Student’s *t*-test was used. To obtain a correlation with the prognosis of head and neck cancer univariate cox analysis was performed using the coxph function in R. The survival curve was drawn by the Kaplan-Meier method for prognostic analysis, and the log-rank test was used to determine the significance of the difference. Statistical significance was set at *p* < 0.05.

## 3 Results

### 3.1 Molecular typing based on ferroptosis-related genes

**FIGURE 1:**
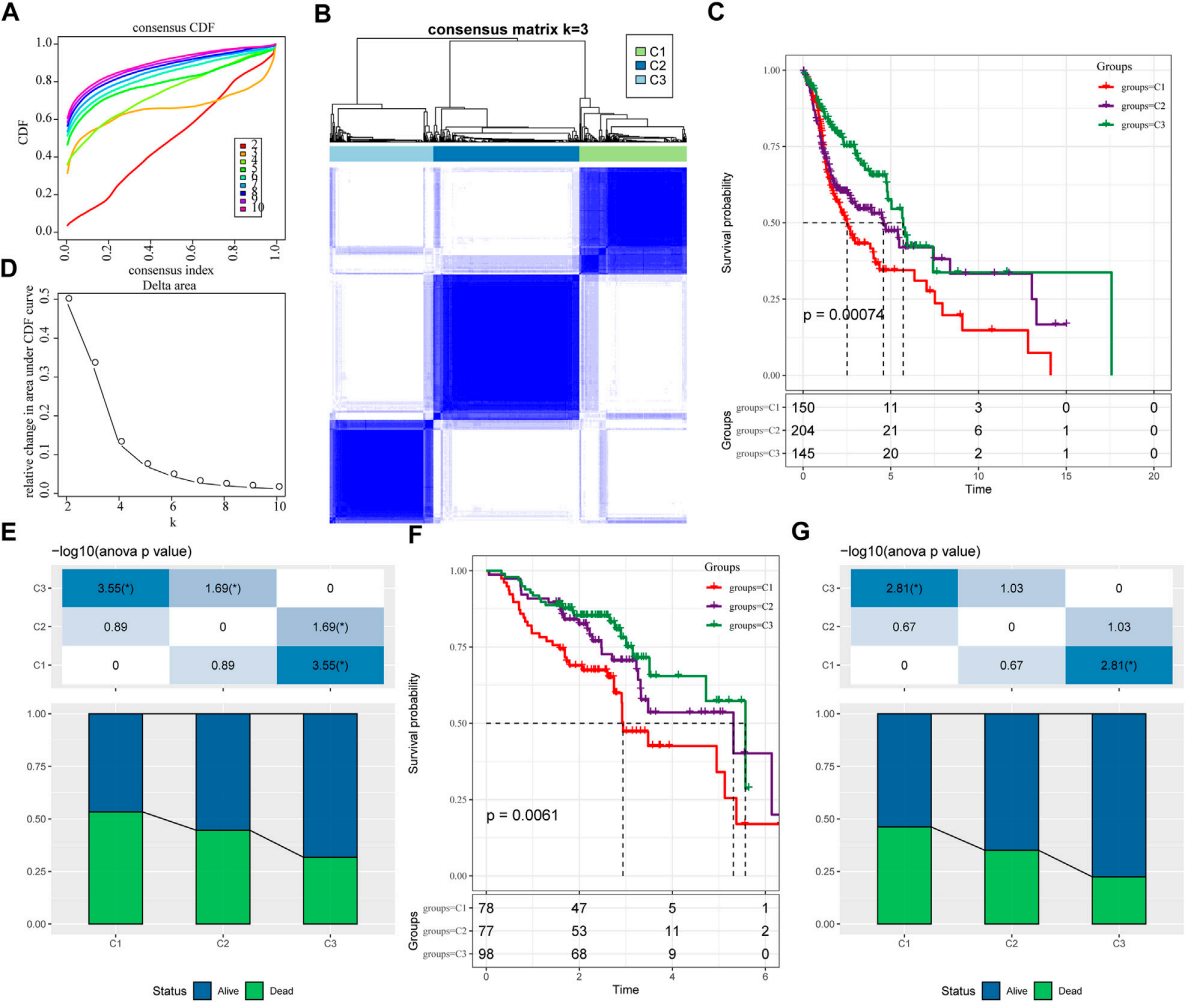
Molecular typing based on ferroptosis-related genes. **(A)** the cumulative distribution function (CDF) curve of TCGA-HNSC cohort sample; **(B)** the CDF Delta area curve of TCGA-HNSC cohort sample. Delta area curve of consensus clustering, indicating the relative change in area under the CDF curve for each category number k compared with k–1. The horizontal axis represents the category number k and the vertical axis represents the relative change in area under CDF curve; **(C)** Consensus heat map of sample clustering when *k* = 3; **(D)** Prognosis of three subtypes Relational Kaplan-Meier curve. **(E)** In TCGA-HNSC Differences in survival status of different subtypes; **(F)** the differences in prognosis of three molecular subtypes in GSE65858 cohort; **(G)** Differences in survival status of different subtypes in GSE65858. The lower half is the proportion, and the upper half is the statistical significance of the distribution difference between the two pairs—log10 (*p*-value).

We extracted the expression levels of ferroptosis-related genes from the expression profile matrix of TCGA-HNSC. Then we got 47 genes after performed a univariate cox analysis using the coxph function in R to obtain a correlation with the prognosis of head and neck cancer (*p* < 0.05) (tcga.ferroptosis.genes.cox.sig.txt). Next we clustered 499 HNSC samples based on the 47 prognostic-related ferroptosis-related genes through ConsensusClusterPlus, determined the optimal number of clusters according to the cumulative distribution function (CDF). And we observed that, the CDF Delta area curve has relatively stable clustering results (Figures 1A,B) when Cluster is selected as 3. Finally we choose *k* = 3 to obtain three molecular subtypes (Figure 1C; tcga.subtype.txt). Further analysis of the prognostic characteristics of these three molecular subtypes, we observed that they have significant prognostic differences as shown in Figure 1D. In general, the C3 subtype has a better prognosis, while the C1 subtype has a poor prognosis. The mortality rate of patients with C1 subtype was significantly higher than that of C3 subtype Figure 1E.

In addition, same method was used to perform molecular typing on the GSE65858 microarray data, and we observed that there were also significant differences in the prognosis of these three types of molecular typing as shown in Figures 1F,G, which is consistent with the training set.

### 3.2 Clinicopathological features between molecular subtypes

**FIGURE 2:**
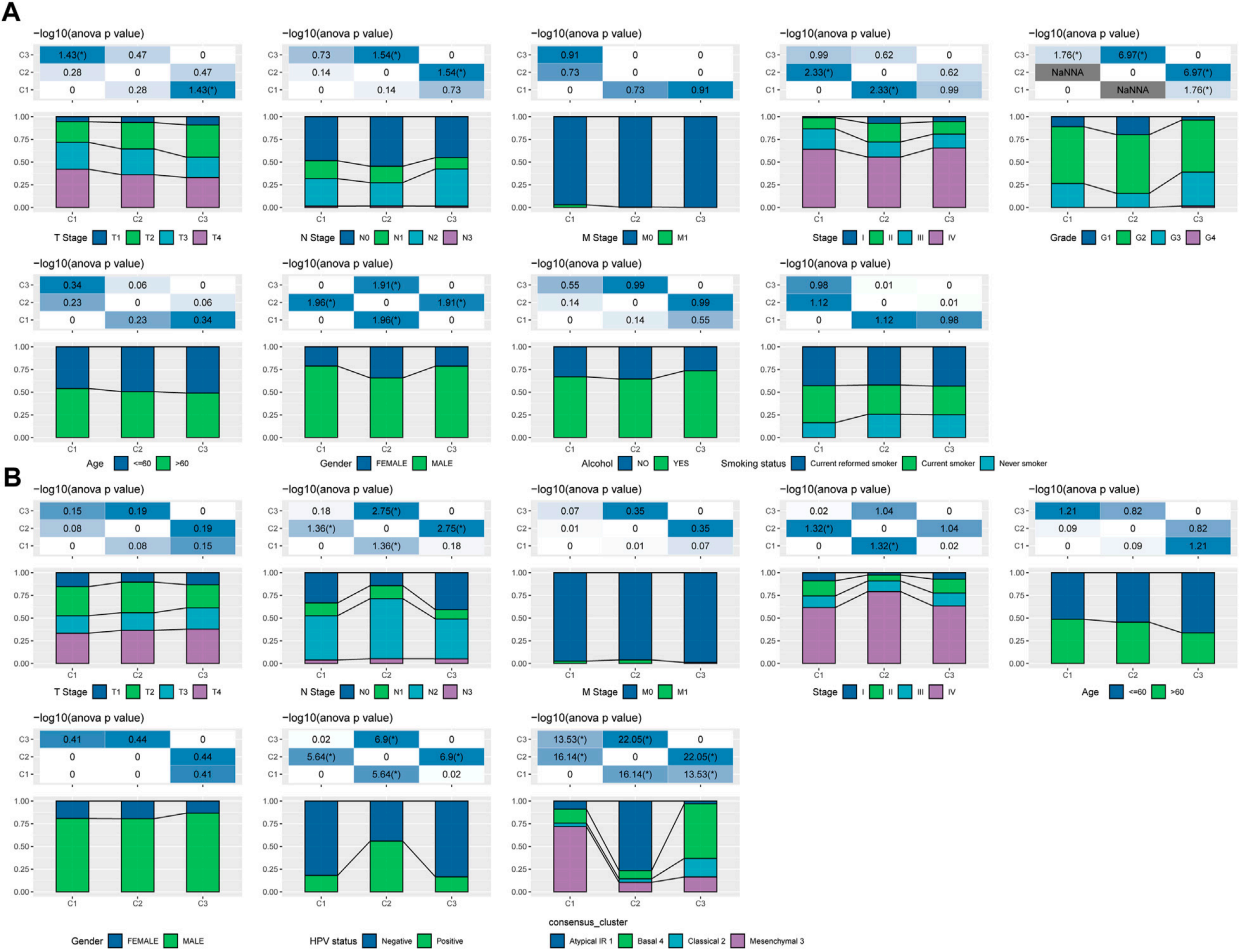
Clinicopathological features between molecular subtypes. **(A)** Clinicopathological characteristics of molecular subtypes of the TCGA-HNSC cohort; **(B)** Clinicopathological characteristics of molecular subtypes of the GSE65858 cohort; the lower half is the proportion, and the upper half is the distribution difference between the pairwise statistically significant-log10 (*p*-value).

We further explored the differences in clinicopathological characteristics between different molecular subtypes in the TCGA-HNSC cohort. Here, we compared the distribution of different clinical features in the three molecular subtypes we defined to see if the clinical features are different in different subtypes. As shown in Figure 2A, it was found that: there were no significant differences between M Stage, age, alcohol consumption and smoking history. However, there were significant differences between C1 and C3 subtypes in terms of T Stage, and between C2 and C3 subtypes in terms of N Stage. And there are significant differences between subtypes C1 and C2 in terms of Stage, and between subtypes C1 and C3 in terms of Grade, and between subtypes C1 and C2 in terms of gender. In addition, we also compared the clinicopathological characteristics of different molecular subtypes in the GSE65858 cohort as shown in Figure 2B.

### 3.3 Mutational signatures between molecular subtypes

**FIGURE 3:**
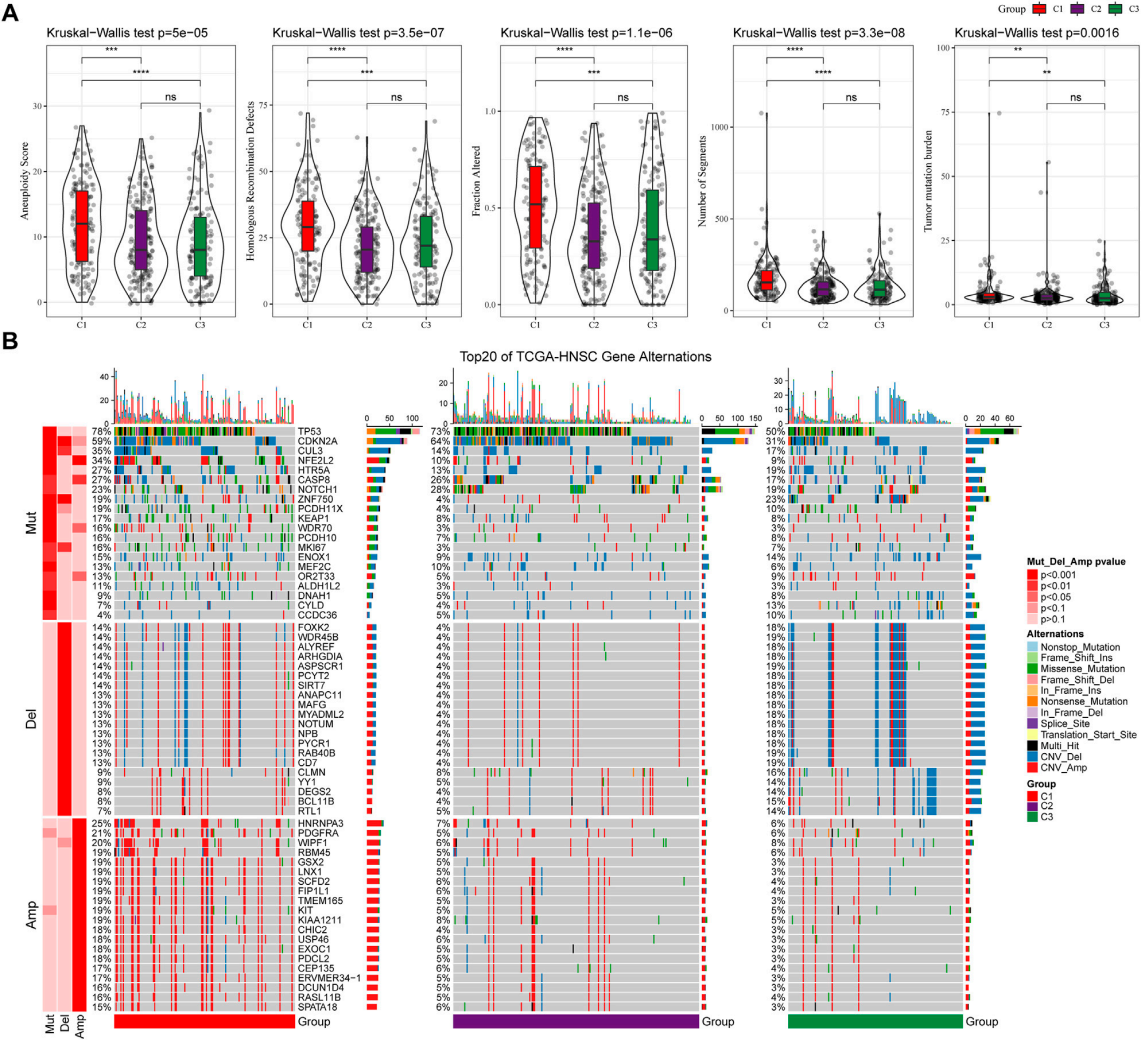
Genomic alterations in molecular subtypes of the TCGA cohort. **(A)** Comparison of Homologous Recombination Defects, Aneuploidy Score, Fraction Altered, Number of Segments and Tumor Mutation Burden among different molecular subtypes in TCGA cohort; **(B)** Somatic mutation and copy number variation analysis of different molecular subtypes in TCGA cohort (Fisher’s test). *p < 0.05; **p < 0.01; ***p < 0.001; and ****p < 0.0001.

We further explored differences in genomic alterations between different molecular subtypes in the TCGA cohort. Compared with the C3 subtype, the C1 subtype showed higher Aneuploidy Score (Kruskal-Wallis test, *p* = 5e-05), Homologous Recombination Defects (Kruskal-Wallis test, *p* = 3.5e-07), Fraction Altered (Kruskal-Wallis test, *p* = 1.1e-06), Number of Segments (Kruskal-Wallis test, *p* = 3.3e-08) and Tumor Mutation Burden (Kruskal-Wallis test, *p* = 0.0016) (Figure 3A). In addition, we also analyzed the correlation between gene mutation and copy number variation and molecular subtype, and found that there was a significant correlation between subtype and gene mutation. Some common genes TP53, CDKN2A, etc. Have higher mutation frequencies in the three subtypes. In terms of copy number variation, the C1 subtype has a higher overall copy number amplification than the C3 subtype, while the C3 subtype has an overall higher copy number deletion than the C1 subtype (Figure 3B).

### 3.4 WGCNA analysis identifies molecular subtype-associated gene modules

**FIGURE 4:**
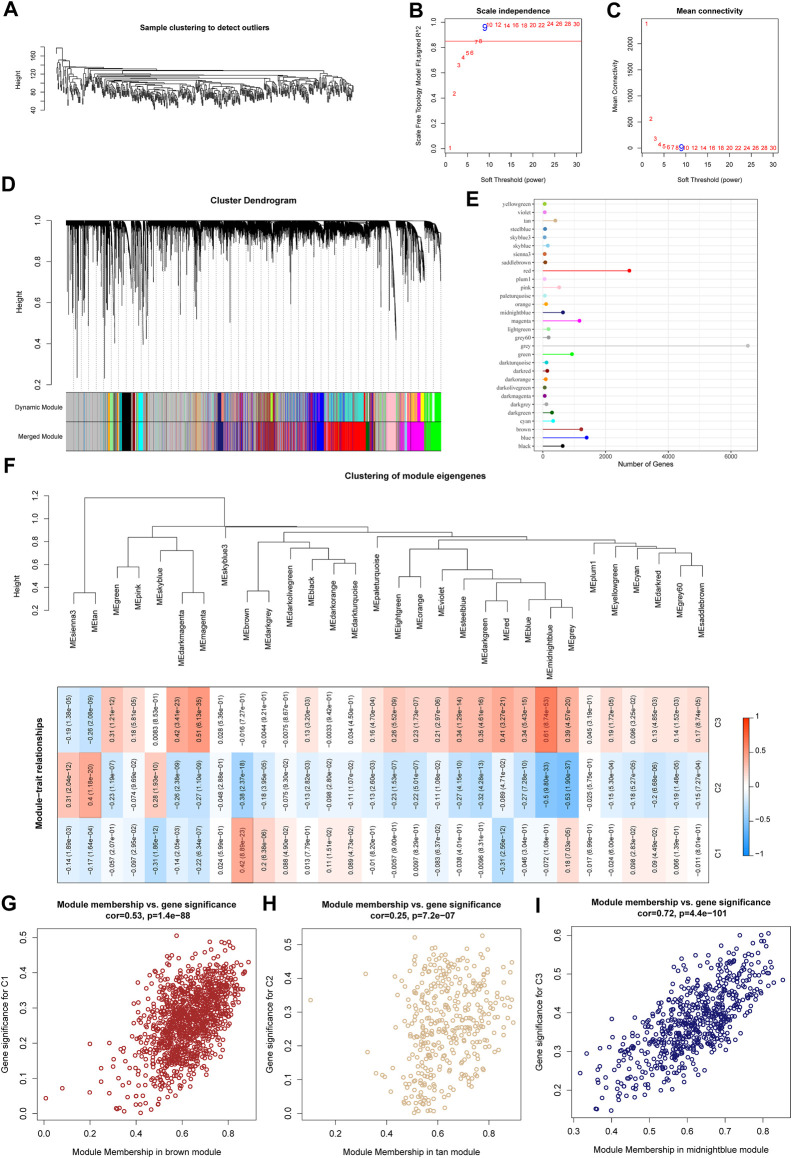
WGCNA analysis identifies molecular subtype-associated gene modules. **(A)** Clustering tree of each sample; **(B)** Analysis of the scale-free fit index for various soft-thresholding powers (β). **(C)** Analysis of the mean connectivity for various soft-thresholding powers. **(D)** Dendrogram of all differentially expressed genes/lncRNAs clustered based on a dissimilarity measure (1-TOM); **(E)** statistics of the number of genes in each module; **(F)** correlation between the module eigenvectors of each module and clinical information; **(G)** Scatter diagram for module membership vs. gene significance for C1 in the brown module; **(H)** Scatter diagram for module membership vs. gene significance for C2 in the tan module; **(I)** Scatter diagram for module membership vs. gene significance for C3 in the middlenightblue module.

We used the R software package WGCNA to identify gene modules related to molecular subtypes. Specifically, samples were firstly clustered to filt for co-expression modules. The clustering results of the samples are shown in Figure 4A. The study shows that the co-expression network conforms to the scale-free network, that is, the logarithm log(k) of a node with a degree of connection k is negatively correlated with the logarithm log (P(k)) of the probability of the node appearing, and the correlation coefficient is greater than 0.85. To ensure that the network is scale-free, we choose β = 9 (Figures 4B,C). The next step is to convert the expression matrix into an adjacency matrix, and then convert the adjacency matrix into a topology matrix. Based on TOM, we use the average-linkage hierarchical clustering method to cluster genes according to the standard of hybrid dynamic shear tree. And set the minimum number of genes for each gene network module is 30. After using the dynamic shearing method to determine the gene modules, we calculate the eigengenes of each module in turn, then perform cluster analysis on the modules, merge the modules with closer distances into a new module, and set height = 0.3, deepSplit = 2, minModuleSize = 30. A total of 39 modules (Figure 4D) were obtained. It should be pointed out that the grey module is a set of genes that cannot be aggregated into other modules. The gene statistics of each module are shown in Figure 4E, and the genes in the modules are shown in tcga.wgcna.module.genes.txt.

Further, we analyzed the correlation of each module with molecular subtypes as shown in Figure 4F.

It can be seen that there is a significant positive correlation between the brown module and the C1 subtype, the tan module and the C2 subtype, and the middlenightblue module and the C2 subtype. There are highly positively correlated between the module membership (MM) and gene significance (GS) of genes within the brown module (r = 0.53, P < 1e-5, Figure 4G), the tan module (*r* = 0.25, *P* < 1e-5, Figure 4H), and the middlenightblue module (*r* = 0.72, *P* < 1e-5, Figure 4I). Further, we used the R software package clusterProfiler to enrich the genes in the brown, tan and middlenightblue modules. The enrichment results are shown in tcga.XXX.enrich.txt.

It was found that the middlenightblue module significantly enriched Estrogen signaling pathway, Ether lipid metabolism, alpha-Linolenic acid metabolism, Linoleic acid metabolism and other pathways (Supplementary Figure S1C), and the gene enrichment results in brown and tan modules are shown in Supplementary Figures S1A,B. The brown, tan and middlenightblue modules with high positive correlation in typing were regarded as the key gene modules related to molecular typing.

### 3.5 Determination of ferroptosis phenotype-related genes

**FIGURE 5:**
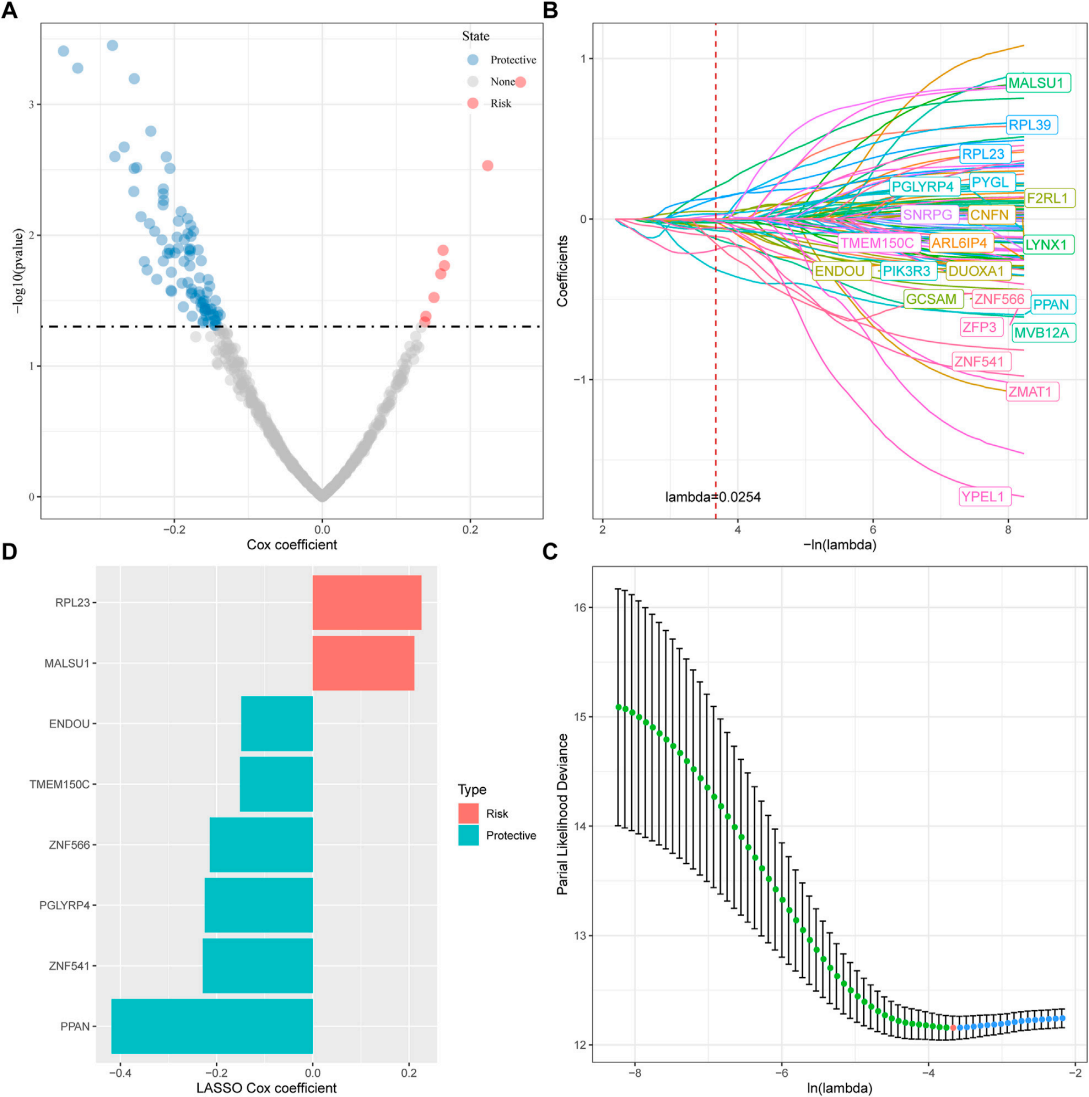
Determination of ferroptosis phenotype-related genes. **(A)** A total of promising candidates were identified through the correlation analysis of the gene expression and the Module Membership; **(B)** The trajectory of each independent variable with lambda; **(C)** Confidence interval under lambda; **(D)** Distribution of LASSO coefficients of the ferroptosis-related prognostic gene signature.

For the genes in the brown, tan and middlenightblue modules identified by WGCNA that are significantly related to molecular subtypes, we first filtered out the genes that are significantly related to the module eigenvectors. Here we select the genes with a correlation coefficient >0.7. After filting, we obtained a total of 540 genes related to the module feature vector (correlation>0.7). The correlation results showed in brown.cor.txt, tan.cor.txt and midnightblue.cor.txt. Further, we aimed at these Univariate COX regression analysis of genes identified 97 genes that had a greater impact on prognosis (*p* < 0.0 5), including 8 risk and 89 protective genes (Figure 5A). Taking it a step further, we compressed these 97 genes in the TCGA-HNSC dataset using lasso regression to reduce the number of genes for the risk model. The Lasso (Least absolute shrinkage and selection operator) method is a compression estimation (Tibshirani, 1996). It obtains a more refined model by constructing a penalty function, so that it compresses some coefficients and sets some coefficients to zero. Therefore, the advantage of subset shrinkage is retained, and it is a biased estimation for processing data with complex collinearity, which can realize the selection of variables at the same time as parameter estimation, and better solve the multicollinearity problem in regression analysis.

Here, we performed lasso cox regression using the R package glmnet. First, the change trajectory of each independent variable is analyzed as shown in Figure 5B. It can be seen that with the gradual increase of lambda, the number of independent variable coefficients tending to 0 also gradually increases. We use 10 -fold cross-validation for the model. Construct and analyze the confidence interval under each lambda as shown in Figure 5C. It can be seen from the figure that the model is optimal when lambda = 0.0254. For this reason, we choose 22 genes when lambda = 0.0254 as the next step target gene. Further, based on the 22 genes in the lasso analysis results, we used stepwise multivariate regression analysis, and the stepwise regression used the AIC Akaike Information Criterion, which considered the statistical fit of the model and the number of parameters used for fitting, stepAIC in the MASS package. The method starts with the most complex model and deletes one variable in turn to reduce the AIC. The smaller the value, the better the model, which means that the model obtains sufficient fit with fewer parameters. Ultimately, we identified 8 genes as ferroptosis-related genes affecting prognosis, as shown in Figure 5D.

### 3.6 Establishment and validation of clinical prognostic model

**FIGURE 6:**
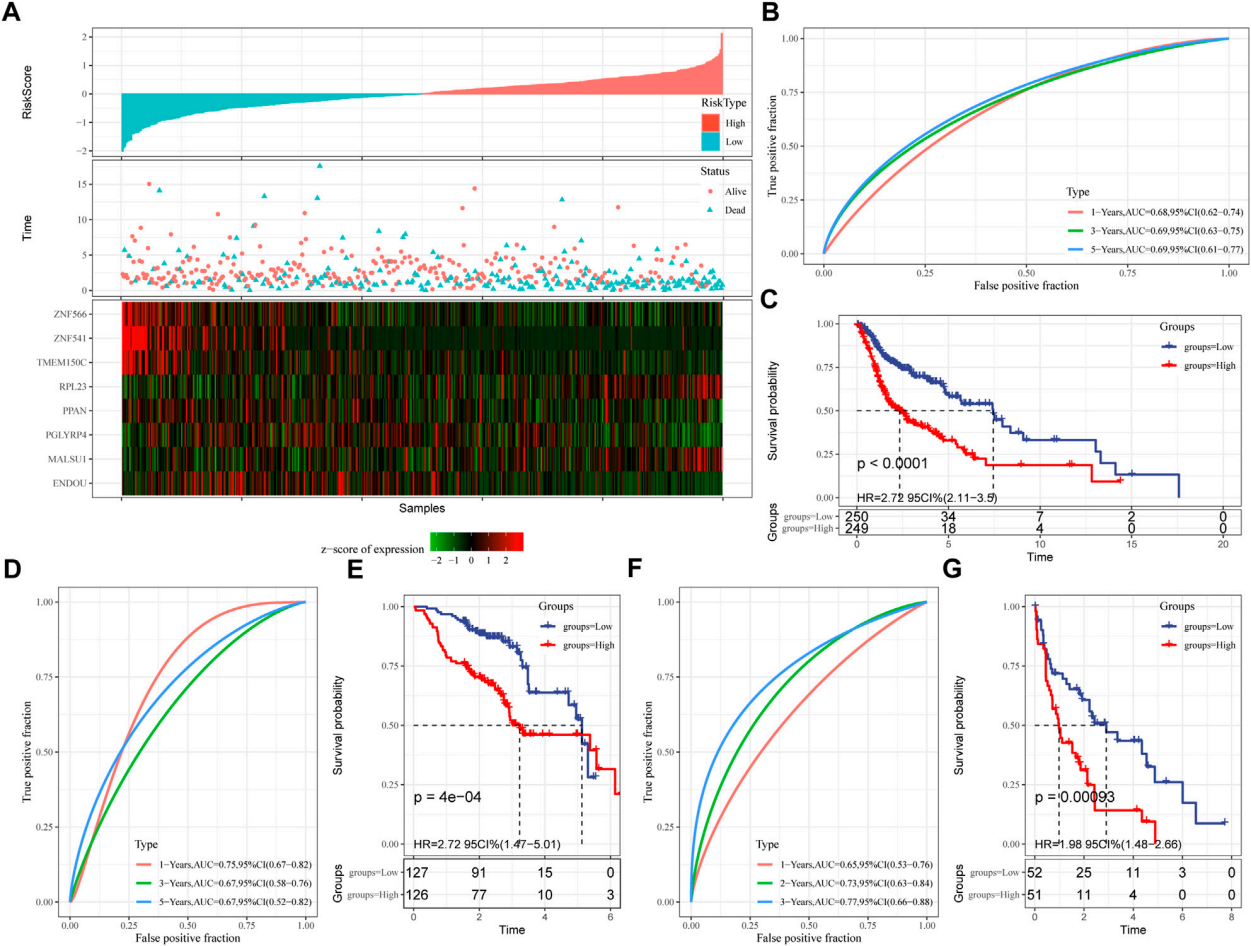
Establishment and validation of clinical prognostic model. **(A)** FPRS in TCGA-HNSC data set, survival time and survival status and expression of ferroptosis-related prognostic genes; **(B)** ROC curve and AUC of FPRS classification in TCGA-HNSC data set; **(C)** FPRS in TCGA- KM survival curve distribution in HNSC dataset; **(D,E)** ROC curve and KM survival curve of FPRS in GSE65858 cohort; **(F,G)** ROC curve and KM survival curve of FPRS in GSE 42743 cohort.

Next, the FPRS for each sample was calculated and normalized according to the formula defined by our sample ferroptosis score. The FPRS distribution of patients in the training set TCGA-HNSC cohort is shown in Figure 6A, which suggests that high FPRS samples have poorer prognosis.The low expression of ZNF566, ZNF541, TMEM150C, PPAN, PGLYRP4, and ENDOU is associated with high risk, which is a protective factor. While the high expression of RPL23 and MALSU1 genes is associated with high risk, which is a risk factor. Further, we used the R software package timeROC to carry out the ROC analysis of the prognostic classification of FPRS. We analyzed the 1-year, 3-year, 5-year prognosis prediction classification efficiency is shown in Figure 6B, from which we can see that the model has a higher area under the AUC line. Finally, we classify the FPRS score greater than 0 as high risk, and the FPRS score less than or equal to 0 as low Risk. And we draw the KM curve, as shown in Figure 6C, there is a very significant difference between the high and low FPRS groups (*p* < 0.0001). 249 samples were divided into the high FPRS group, and 250 samples were divided into the low FPRS group. Patients with higher FPRS exhibited worse overall survival in the training cohort (Figure 6C). To confirm the robustness of ferroptosis-related gene signature clinical prognostic model predictions, we performed validation in two independent head and neck cancer cohorts, and we calculated the FPRS scores of patients in the same way. Seeing that in the validation cohort we observed similar results to the training set, with high FPRS having a worse prognosis and low FPRS having a better prognosis (Figures 6D–G).

### 3.7 FPRS scores on different clinicopathological features and different molecular subtypes

**FIGURE 7:**
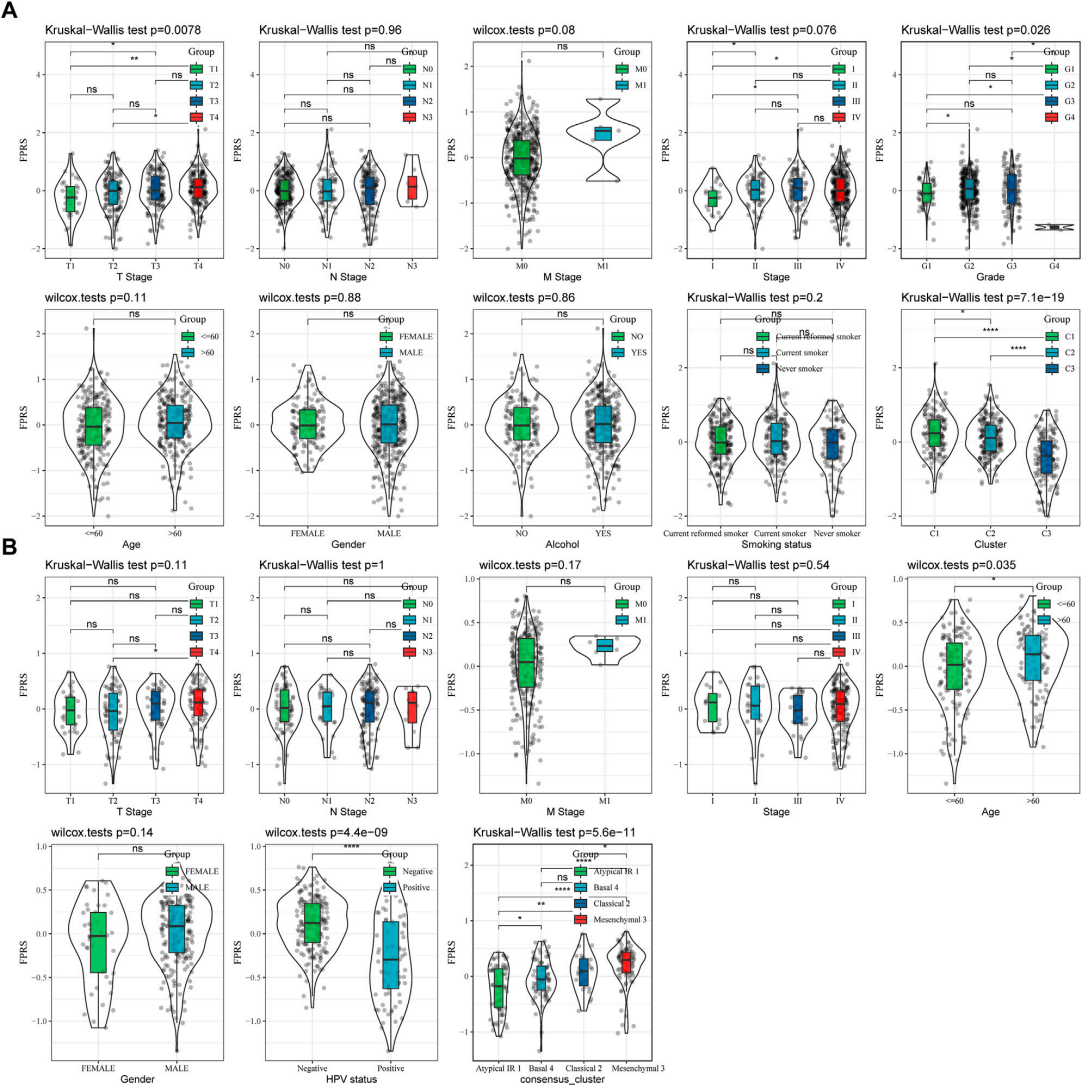
FPRS scores on different clinicopathological features and different molecular subtypes. **(A)** Differences in FPRS between different clinicopathological groups in TCGA-HNSC cohort; **(B)** Differences in FPRS between different clinicopathological groups in GSE65858 cohort.

By comparing the distribution of FPRS among clinicopathological groups, we found that higher TNM stage means higher FPRS (Figure 7A). And we also found no significant difference in FPRS between ages (Figure 7A). At the same time, we compared the differences of FPRS among molecular subtypes and found that the FPRS of the C1 subtype with poor prognosis had the highest FPRS score, while the C3 molecular subtype with better prognosis had the lowest FPRS (Figure 7B).

### 3.8 Mutation signatures between FPRS groups

**FIGURE 8:**
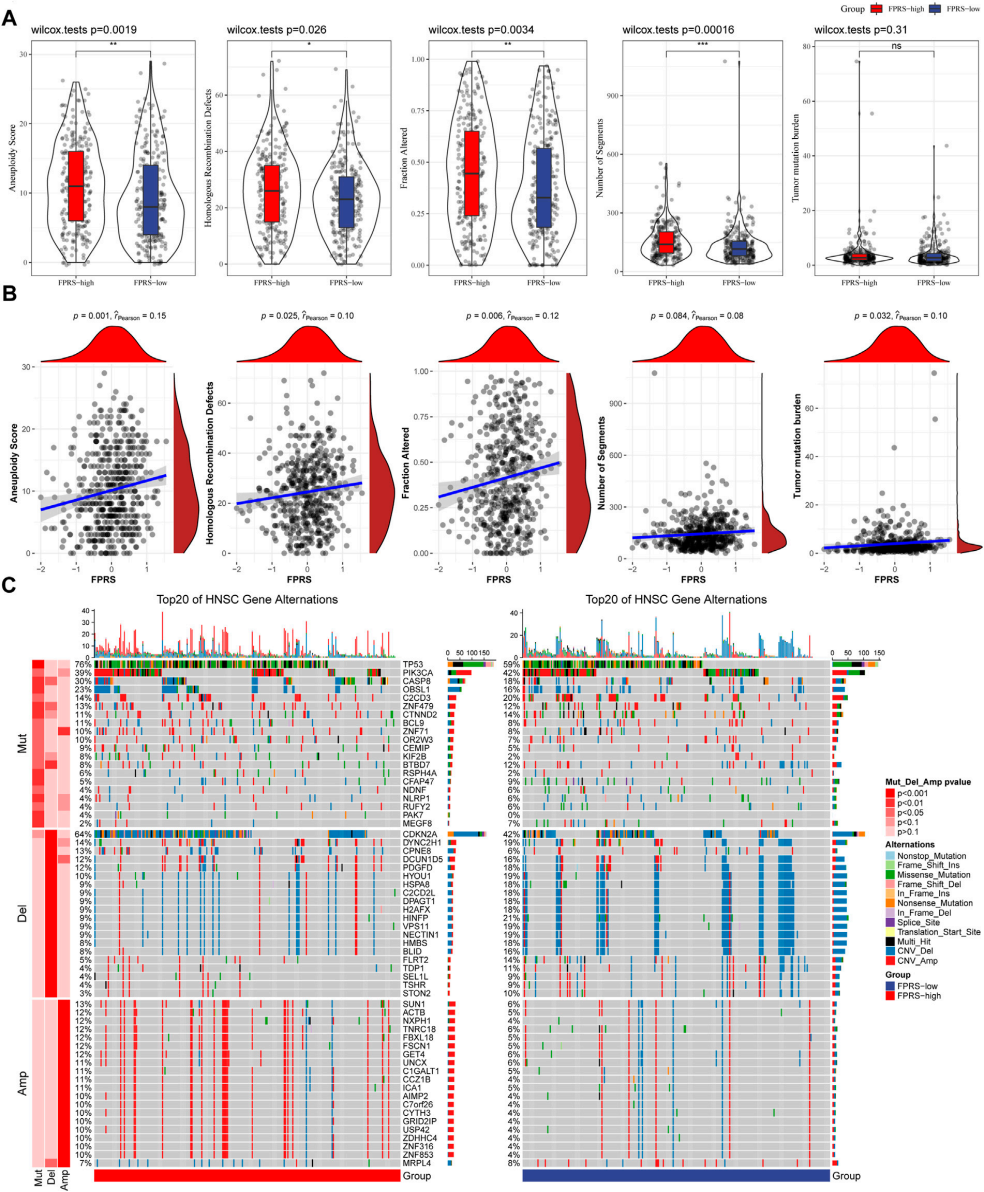
Differences inFPRS groupings of the TCGA cohort. **(A)** Compare the differences in Homologous Recombination Defects, Aneuploidy Score, Fraction Altered, Number of Segments and Tumor mutation burden in different FPRS groups of the TCGA cohort; **(B)** FPRS and Homologous Recombination Defects, Aneuploidy Score, Fraction Altered, Number of Segments and Tumor mutation Correlation between burden; **(C)** Somatic mutation and copy number variation analysis (Fisher test) of FPRS groupings in the TCGA cohort. *p < 0.05; **p < 0.01; ***p < 0.001; and ****p < 0.0001.

We further explored differences in genomic alterations between different FPRS subgroups in the TCGA cohort. Compared with the FPRS-low group, the FPRS-high group showed higher Aneuploidy Score (wilcox. test, *p* = 0.0019), Homologous Recombination Defects (wilcox. test, *p* = 0.026), Fraction Altered (wilcox. test, *p* = 0.0034), and Number of Segments (wilcox. test, *p* = 0.00016) (Figure 8A). At the same time, we also analyzed the correlation between FPRS and Homologous Recombination Defects, Aneuploidy Score, Fraction Altered, Number of Segments, and Tumor mutation burden, and found that FPRS was significantly positively correlated with Aneuploidy Score (*p* = 0.001), Fraction Altered (*p* = 0.006), and Tumor Mutation Burden (*p* = 0.032) (Figure 8B). In addition, we also analyzed the correlation between gene mutation and copy number variation and molecular subtype, and found that there was a significant correlation between subtype and gene mutation. The common TP53 was mutated at a higher frequency in both subtypes. In terms of copy number variation, the copy number deletion in the FPRS-low group was generally higher than that in the FPRS-high group, while the copy number amplification was lower than that in the FPRS-high group (Figure 8C).

### 3.9 Path characteristics between FPRS packets

**FIGURE 9:**
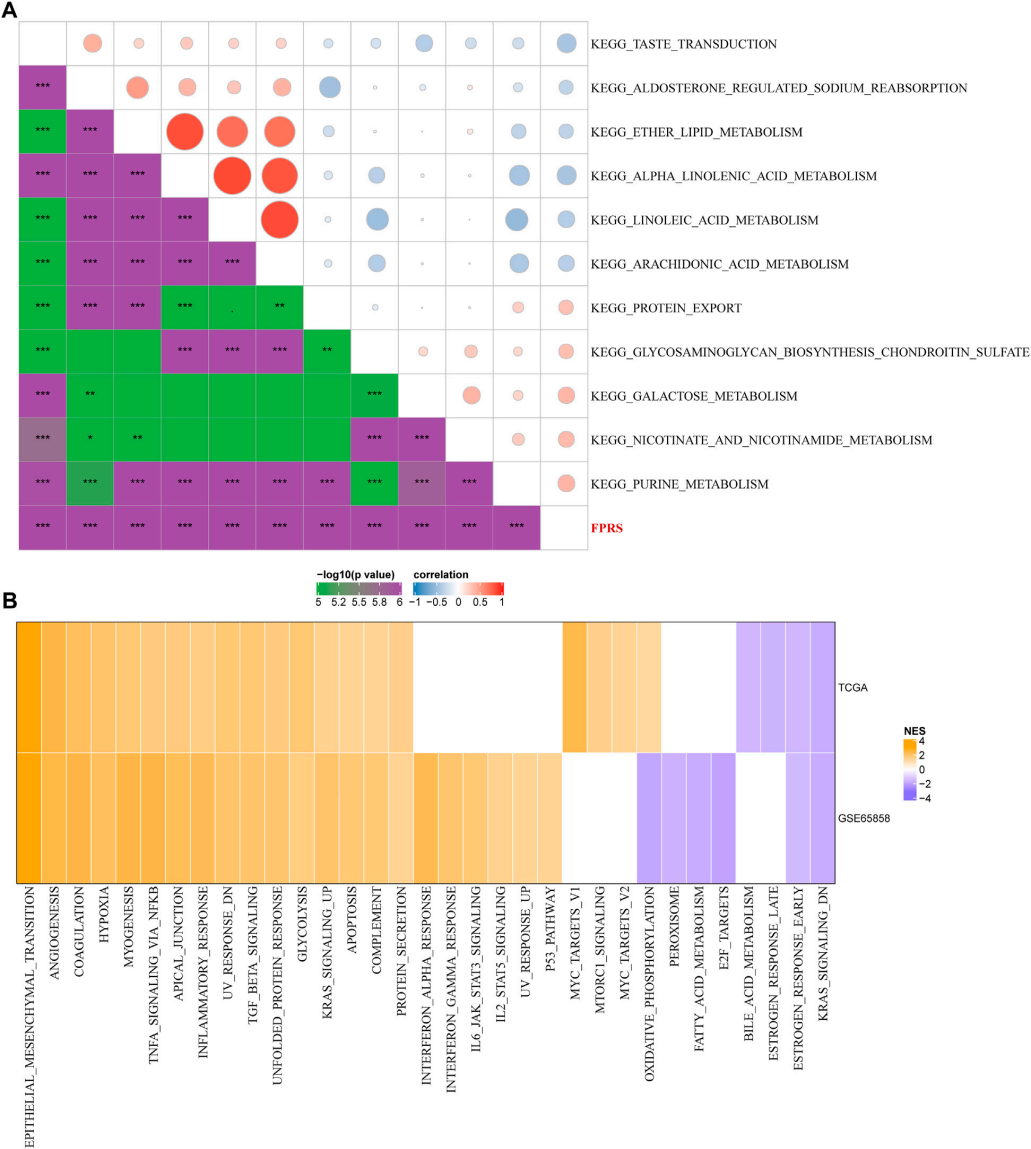
Path characteristics between FPRS packets. **(A)** Correlation analysis results between KEGG pathways with FPRS correlation greater than 0.3 and FPRS; **(B)** a heatmap demonstrating normalized enrichment scores (NESs) of Hallmark pathways calculated by comparing FPRS-high with FPRS-low (with a false discovery rate (FDR) of < 0.05).

In order to observe the relationship between FPRS and biological function in different, we selected the gene expression profiles corresponding to the head and neck cancer samples in the TCGA-HNSC cohort using the R software package GSVA to perform a single-sample GSEA analysis (ssgsea). The ssGSEA score of each function corresponding to each sample is obtained after calculating the different functions of each sample. And the correlation between these functions and FPRS is further calculated, and the function with a correlation greater than 0.4 is selected (Figure 9A). It can be seen that 13 of pathways was positively correlated with the FPRS of the samples, and 8 pathways were negatively correlated with the FPRS. Among them, the metabolism-related pathway KEGG_PROTEIN_EXPORT, KEGG_ GLYCOSAMINOGLYCAN_ BIOSYNTHESIS_CHONDROITIN_SULFATE, KEGG_GALACTOSE_METABOLISM, KEGG_NICOTINATE_AND_NICOTINAMIDE_ METABOLISM, KEGG_PURINE_METABOLISM showed a significant positive correlation with FPRS. Next, we analyzed whether there are differentially activated pathways in different FPRS groupings. To identify these pathways, we performed Gene Set Enrichment Analysis (GSEA) using all candidate gene sets in the Hallmark database [PMID: 26771021], where we defined FDR < 0.05 as significant enrichment as shown in Figure 9B. It can be seen that compared with PFRS-low in TCGA-HNSC cohort, 20 pathways were activated in PFRS-high, 4 pathways were inhibited, and 28 pathways were significantly enriched in GSE65858 cohort. Overall, the activated pathways in the PFRS-high group mainly included immune-related pathways such as INFLAMMATORY_RESPONSE, COMPLEMENT, etc., and the invasion-related pathways such as EPITHELIAL_MESENCHYMAL_TRANSITION, ANGIOGENESIS, TNFA_SIGNALING_*VIA*_NFKB, etc. were significantly enriched in the FPRS-high group as shown in Figure 8B. Overall, the activation of immune-related pathways and the activation of invasion -related pathways in the FPRS-high group may be potential factors for the poor prognosis of FPRS-high.

### 3.10 Immune signatures between FPRS subgroups

**FIGURE 10:**
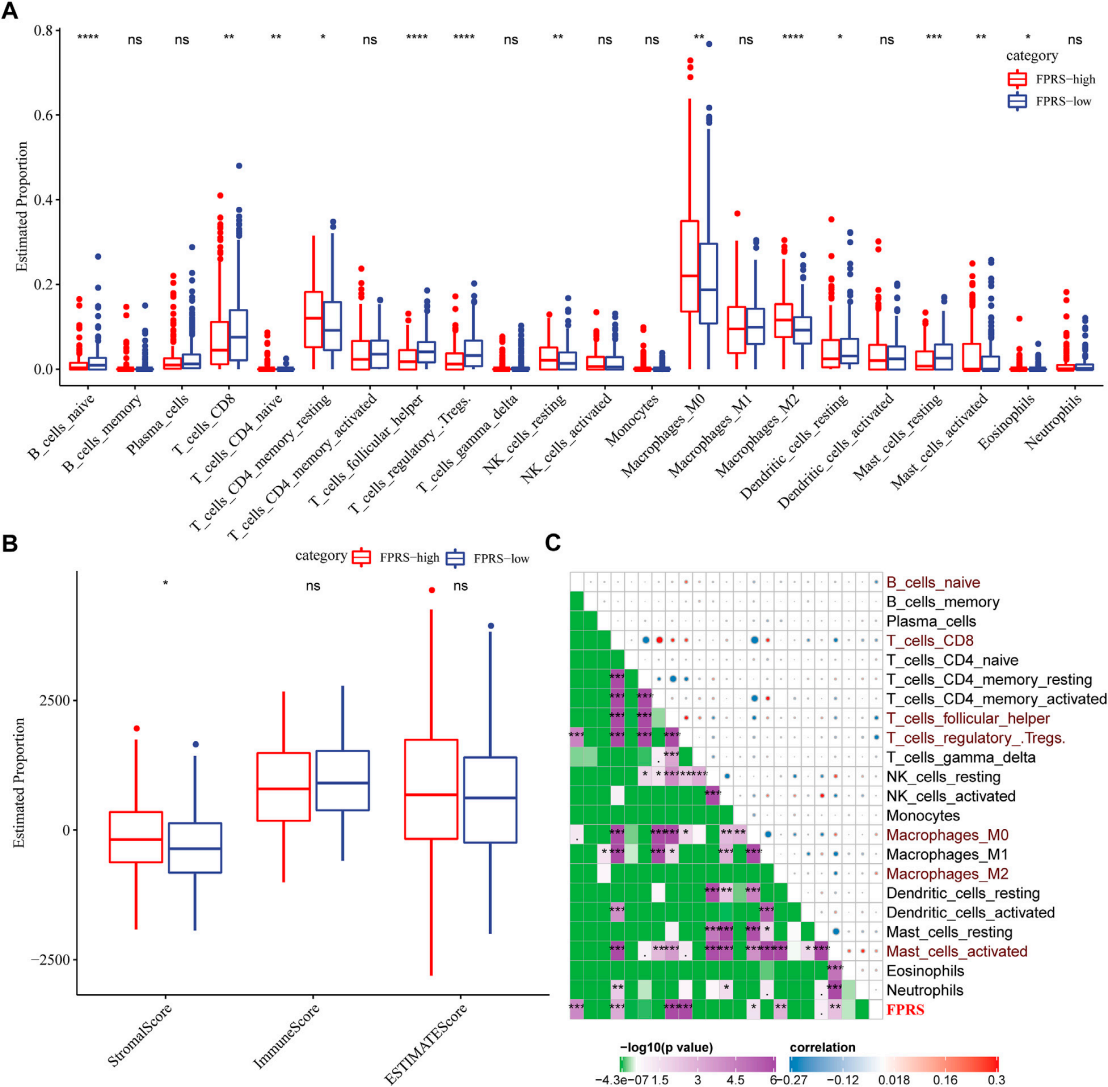
Immune signatures between FPRS subgroups. **(A)** The proportion of immune cell components in the TCGA- HNSC cohort; **(B)** The proportion of immune cell components in the TCGA-HNSC cohort calculated by ESTIMATE software; **(C)** The correlation analysis of 22 immune cell components and FPRS.

To further elucidate the differences in the immune microenvironment of patients in the FPRS cohort, we assessed the extent of immune cell infiltration in patients in our TCGA-HNSC cohort by using the expression levels of gene markers in immune cells, we first employed CIBERSORT to calculate 22 The relative abundance of immune cells is shown in Figure 10A, and it can be observed that B_cells_naive, T_cells_CD8, T_cells_follicular_helper, T_cells_regulatory_.Tregs. are significantly enriched in the FPRS-low group. At the same time, we also used ESTIMATE to evaluate the infiltration of immune cells, as shown in Figure 10B. It can be seen that the ImmuneScore in the FPRS-low group was slightly higher than that in the FPRS-high group, with higher immune cell infiltration. Further, we analyzed the relationship between FPRS and 22 immune cell components, and found that FPRS was significantly negatively correlated with B_cells_naive, T_cells_CD8, T_cells_follicular_helper, T_cells_regulatory_.Tregs., and significantly positively correlated with Macrophages_M2, Mast_cells_activated.

### 3.11 Differences in immunotherapy/chemotherapy between FPRS groups

**FIGURE 11:**
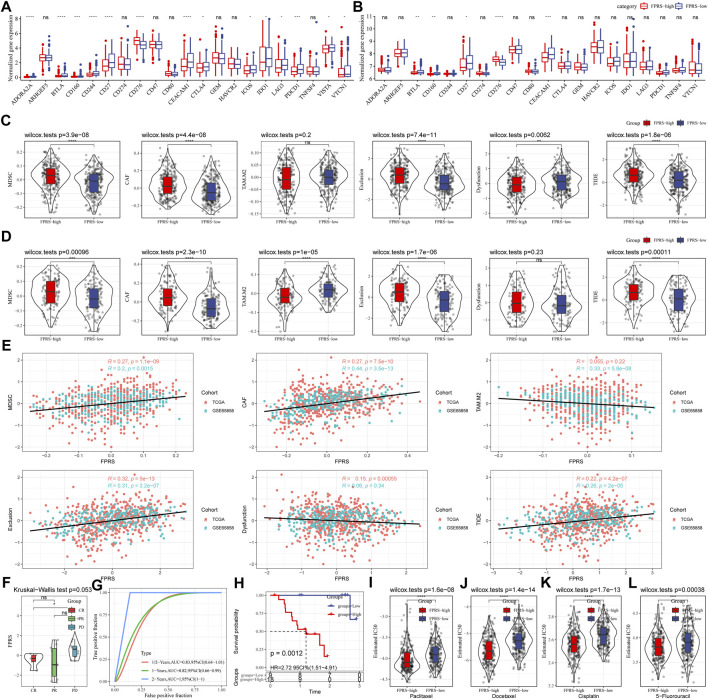
Differences in immunotherapy/chemotherapy between FPRS groups. **(A)** Differentially expressed immune checkpoints between different groups in the TCGA-HNSC cohort; **(B)** Differentially expressed immune checkpoints between different groups in the GSE65858 cohort; **(C)** TIDE between different groups in the TCGA-HNSC cohort Differences in analysis results; **(D)** Differences in TIDE analysis results between different groups in the GSE65858 cohort; **(E)** Correlation between TIDE scores and FPRS scores; **(F)** Differences in FPRS for different clinical response status to immunotherapy in the GSE78220 cohort; **(G)** ROC curve and AUC of FRPS classification; **(H)** KM survival curve distribution of FPRS; **(I–L)**: The box plots of the estimated IC50 for Paclitaxel, Docetaxel, Cisplatin and 5–Fluorouracil in TCGA—HNSC.

Further, we analyzed whether there were differences in response to immunotherapy between the FPRS groups. First, we compared whether there are differences in the expression of immune checkpoints between FPRS groups. Here, our immune checkpoints come from the database HisgAtlas [PMID: 31725860]. The results are shown in Figures 11A,B. Most immune checkpoint genes were differentially expressed in FPRS groups. Combining the expression of immune checkpoints in the two cohorts, we found that immune checkpoints such as BTLA, CD160, CD27, and CEACAM1 were significantly up-regulated in the FPRS-low group as shown in Figures 11A,B. Further, we analyzed the differences in immunotherapy among different FPRS groups. Here, we used TIDE (http://tide.dfci.harvard.edu/) software to assess the potential clinical effects of immunotherapy in our defined FPRS high and low groups. The higher the TIDE prediction score, the higher the possibility of immune escape, and the lower the possibility of patients benefiting from immunotherapy. As shown in Figure 11C, we can find that the FPRS-high group has the highest TIDE score in the TCGA-HNSC cohort. It suggesting that the FPRS-high group has a higher possibility of immune escape and is less likely to benefit from immunotherapy. At the same time, we also compared the differences in the predicted T cell dysfunction score and T cell exclusion score among different metabolite subtypes in the TCGA-HNSC cohort, as shown in Figure 11C. And it can be found that the FPRS-high subtype has the highest MDSC, CAF and Exclusion. A similar result was observed in the GSE65858 cohort as shown in Figure 11D.

In addition, we also analyzed the correlation between FPRS score and TIDE score, and found that there was a significant positive correlation between FPRS score and TIDE score (Figure 11E). Further, we included a set of immunotherapy data GSE78220 (anti-PD-1), and calculated the FPRS scores of the samples in the same way, and found that there were differences in the FPRS scores of different clinical response states of immunotherapy in the GSE78220 data set. The FPRS score of patients with response to immunotherapy was lower than that of patients with PD and SD response status (Figure 11F). At the same time, we also found that our clinical prognostic model had stable performance in the two immunotherapy cohorts, with better classification efficiency for prognosis prediction at 6 months, 1 year, and 2 years and with higher AUC area below the line (Figure 11G). There was a significant difference in prognosis between the high and low FPRS groups (Figure 11H). In addition, we also analyzed the response of FPRS subgroups to traditional chemotherapy drugs Paclitaxel, Docetaxel, Cisplatin and 5-Fluorouracil in the TCGA-HNSC cohort, and found that FPRS-high was more sensitive to these four drugs as shown in Figures 11I–L.

### 3.12 FPRS combined with clinicopathological features to further improve prognostic models and survival prediction

**FIGURE 12:**
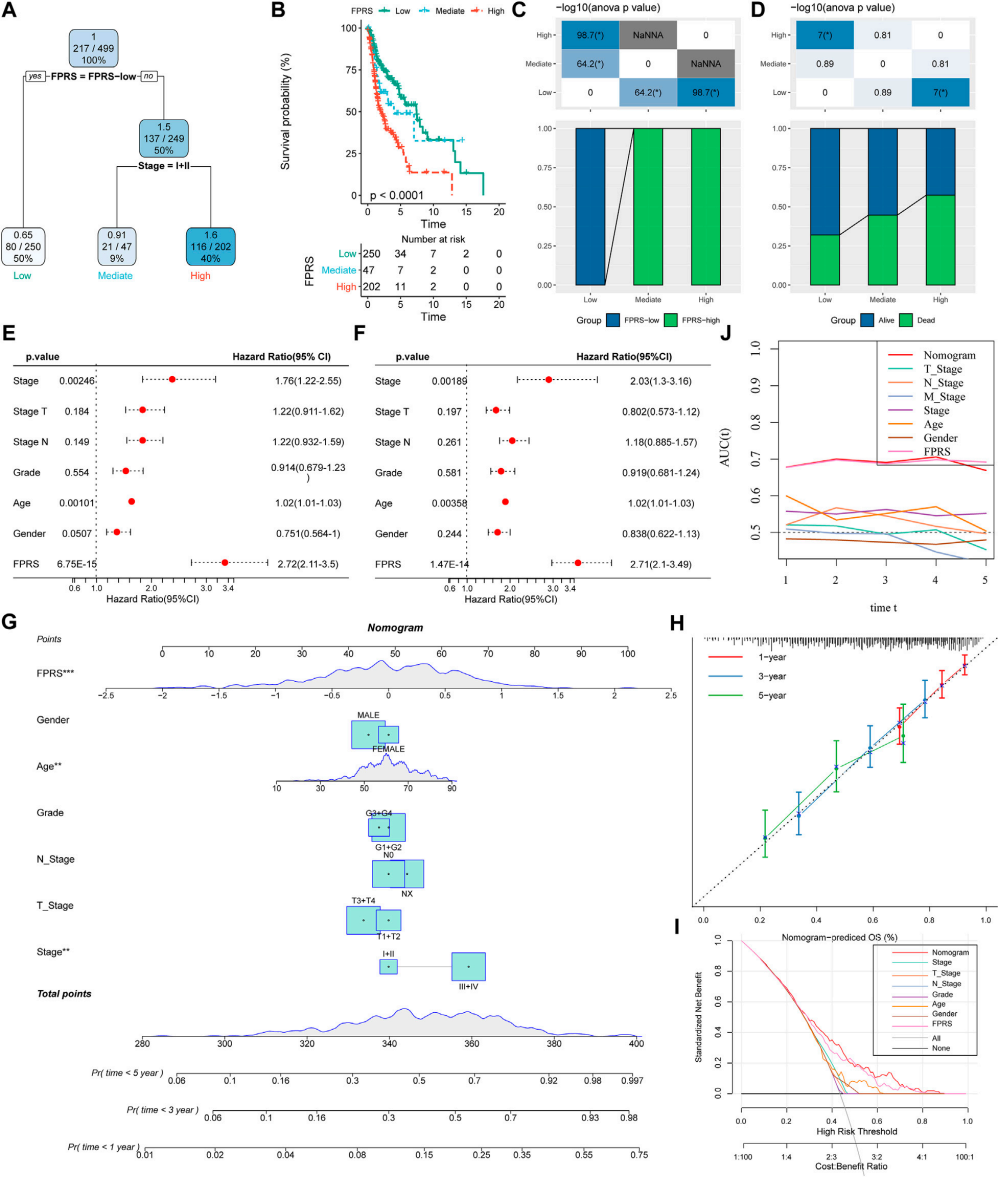
FPRS combined with clinicopathological features to further improve prognostic models and survival prediction. **(A)** Patients with full-scale annotations including FPRS, stage, gender and age were used to build a survival decision tree to optimize risk stratification; **(B)** Significant differences of overall survival were observed among the three risk subgroups; **(C,D)** Comparative analysis between different groups; **(E,F)** univariate and multivariate Cox analysis of FPRS and clinicopathological characteristics; **(G)** nomogram model; **(H)** 1, 3, and 5-year calibration curve of nomogram; **(I)** Decision curve of nomogram; **(J)** Compared with other clinicopathological features, the nomogram exhibited the most powerful capacity for survival prediction.

Here, we constructed a decision tree based on age, gender, T stage, N Stage, Stage, Grade, and FPRS of head and neck cancer patients in the TCGA-HNSC cohort. The results showed that only FPRS and Stage were left in the decision tree and determined three distinct risk subgroups (Figure 12A). Among them, FPRS is the most powerful parameter, followed by the stage. Here, we defined patients with low FPRS as the low risk group, while the intermediate risk group was labeled with low FPRS and Stage low and the high risk group was labeled with high FPRS and Stage high. There were significant differences in overall survival between the three risk subgroups as shown in Figure 12B. The patients in the high risk group and the intermediate risk group were all FPRS-high patients, while the patients in the low risk group were all FPRS-low patients as shown in Figure 12C. In addition, we also found that the high risk group and the intermediate risk group were the proportion of patients in the Dead group was significantly higher than that in the intermediate risk and low risk groups as shown in Figure 12D. Univariate and multivariate Cox regression analysis of FPRS and clinicopathological features showed FPRS to be the most significant prognostic factor (Figures 12E,F).

In order to quantify the risk assessment and survival probability of patients with head and neck cancer, we combined FPRS and other clinicopathological features to establish a nomogram as shown in Figure 12G. From the model results, it can be seen that FPRS has the greatest impact on survival rate prediction. Further, we use the calibration curve (Calibration curve) to evaluate the prediction accuracy of the model, as shown in Figure 12H. It can be observed that the predicted calibration curves of the three calibration points at 1, 3, and 5 years are nearly coincident with the standard curve, which suggests that the nomogram has a good Predictive performance. In addition, we also used DCA (Decision curve) to evaluate the reliability of the model. It can be observed that the benefits of FPRS and nomogram are significantly higher than those of extreme curves. Compared with other clinicopathological characteristics, both nomogram and FPRS show the strongest survival predictive power is shown in Figures 12I,J.

### 3.13 Validation of the 4 ferroptosis-related genes in HNSC tissues

**FIGURE 13:**
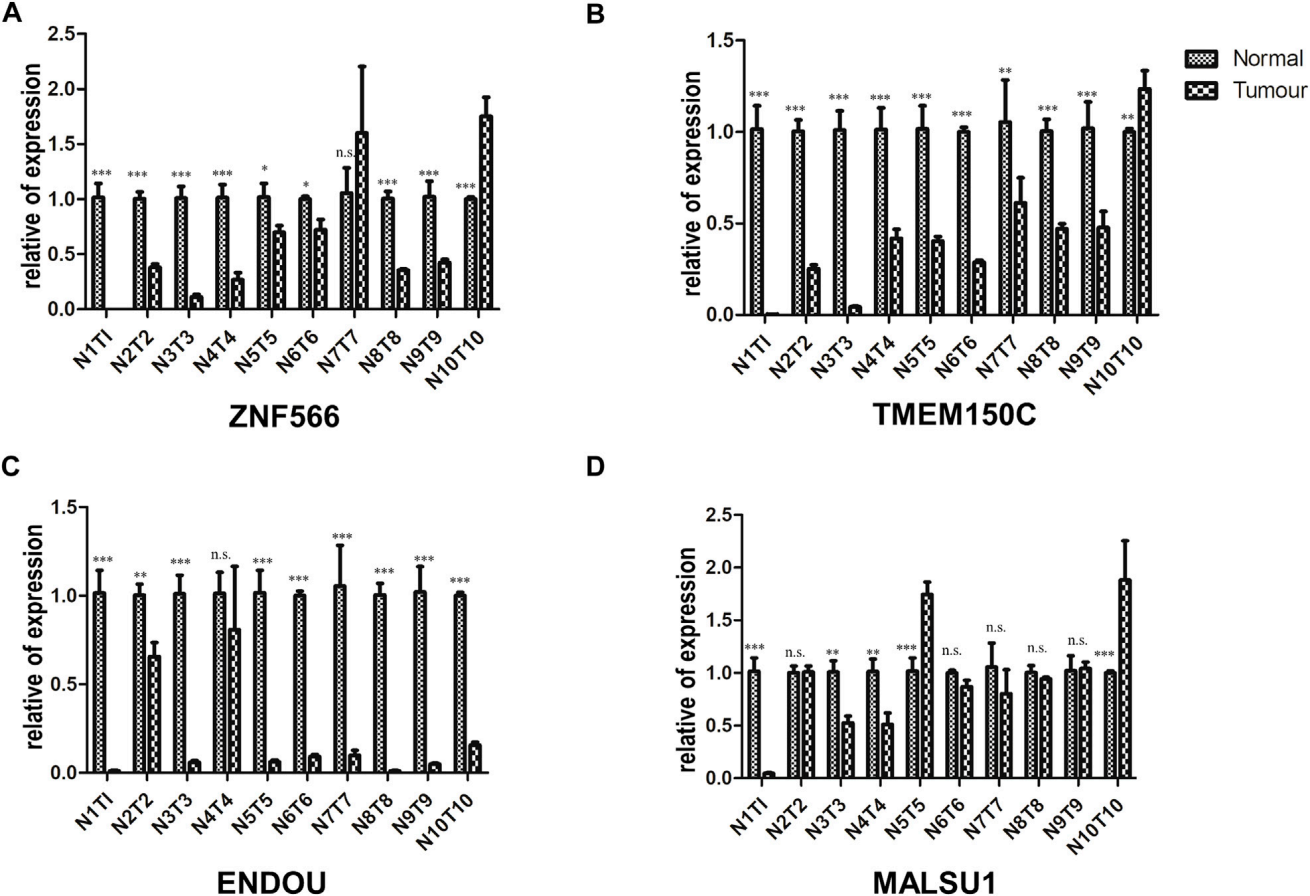
Validation of the 4 ferroptosis-related genes in HNSC tissues. RT-qPCR detecting the mRNA expression levels of **(A)** ZNF566, **(B)** TMEM150C, **(C)** ENDOU, **(D)** MALSU1 in the tumor tissue and adjacent tissue of HNSCC patients and normal tissues HNSCC patients*p < 0.05; **p < 0.01; ***p < 0.001.

We validated the mRNA expression levels of the 4 ferroptosis-related genes (ZNF566, TMEM150C, ENDOU, MALSU1) in the tumor tissue and adjacent tissue of 10 HNSCC patients by RT-qPCR. The results showed that in most patients ZNF566 (8/10, 80%, Figure 13A), TMEM150C (9/10, 90%, Figure 13B), ENDOU (8/10, 80%, Figure 13C) were all significantly decrease in HNSCC tissues than in normal tissues (*p* < 0.005). There was no statistical difference in the expression of MALSU1 (Figure 13D).

## 4 Discussion

Identification of key biomarkers to assess tumor prognosis raises implications for early tumor diagnosis, treatment regimen selection, and cancer prevention (Economopoulou et al., 2019). The prognosis of patients with head and neck cancer is related to many factors, such as age, smoking, gender, TNM stage, stage, drug sensitivity, immune cell infiltration, etc (Zhang et al., 2020; Zhang et al., 2021; Yao et al., 2022). In this work, we got 47 ferroptosis-related genes to obtain a correlation with the prognosis of head and neck cancer patients from the FerrDb database. We identified three stable molecular subtypes (C1, C2, C3) through these genes in TCGA-HNSC cohort. These subtypes are also validated in GSE65858 microarray data. These subtypes did not differ significantly in M Stage, age, alcohol consumption and smoking history. C1 subtype, which has a worst prognosis, has higher tumor mutational burden than other subtypes. In many cancer types, high tumor mutational burden (TMB) is associated with longer survival after immune checkpoint inhibitor (ICI) therapy. While in patients not receiving ICI therapy, higher TMB is associated with worse survival (Valero et al., 2021). Some common genes TP53, CDKN2A, etc. Have higher mutation frequencies in the three subtypes. Recent study had reported a variant of p53 at codon 47 (S47) found in African-descent populations, which alters the ability of p53 to induce cell death and suppress tumor formation (Jennis et al., 2016). This variant leads to accumulation of GSH and CoA (Leu et al., 2019).

To evaluate head and neck cancer samples we construction of the FPRS scoring system. Patients with higher FPRS exhibited worse overall survival. We observed the similar results after validating two independent head and neck cancer cohorts, which confirmed the robustness of ferroptosis-related gene signature clinical prognostic model predictions. The FPRS of the C1 subtype with poor prognosis had the highest FPRS score, while the C3 molecular subtype with better prognosis had the lowest FPRS. FPRS-high group has a higher possibility of immune escape and is less likely to benefit from immunotherapy, which lead to worse prognosis.

The low expression of ZNF566, ZNF541, TMEM150C, PPAN, PGLYRP4, and ENDOU is considered high risk, predicts a worse prognosis. This is generally consistent with the trend we showed in our clinical sample validation. In most validated samples ZNF566, TMEM150C, ENDOU were all significantly decrease in tumor tissues than in normal tissues. ZNF566 plays a central role in heart regeneration and repair through epithelial to mesenchymal transitions (EMT) (Xin et al., 2013; von Gise and Pu, 2012). Considering that EMT is highly correlated with tumor metastasis, it has an important impact on prognosis. CircZNF566 is highly expressed in hepatoma cells and tissues and positively correlated with poor prognosis (Li et al., 2020). This suggests a complex role of ZNF566 in different tissues. ZNF541 mediates chromatin remodeling and is associated with histone hypoacetylation, normally expressed in germ cells (Choi et al., 2008). The U.S. Food and Drug Administration approved histone deacetylase inhibitors for PTCL (O'Connor et al., 2014). TMEM150C (Tentonin 3) was identified as a cation channel activated by mechanical stimulation with unique slow inactivation kinetics and is a molecular component that helps sense changes in dynamic arterial pressure in baroreceptors (Hong et al., 2016; Lu et al., 2020). In pancreatic β-cells TMEM150C is highly expressed, which regulates glucose-stimulated insulin secretion *in vivo* (Wee et al., 2021). Human PPAN localizes to the nucleolus and mitochondria, and PPAN knockdown triggers a p53-independent nucleolar stress response that ultimately leads to mitochondrial apoptosis (Olausson et al., 2012). PPAN knockdown is also associated with mitochondrial damage and stimulation of autophagy (Dannheisig et al., 2019). ENDOU (PP11) was detected in 66.7% of analyzed mucinous cystadenocarcinomas, 57.1% of serous cystadenocarcinomas, but not in normal ovaries, 47% of breast cancers and 38% of all testicular and gastric cancers (Inaba et al., 1980; Inaba et al., 1981; Inaba et al., 1982).

In this work, we constructed a scoring Ferroptosis-related prognostic model that can well reflect risk and positive factors for prognosis in patients with head and neck squamous cell carcinoma. It can be used to guide individualized adjuvant therapy and chemotherapy for patients with head and neck cancer. Therefore, it has a good survival prediction ability and provides an important reference for clinical treatment. Undoubtedly, our prognostic model is limited by the use of public databases. More *in vitro* and *in vivo* studies and clinical research were need to validate the clinical utility of this model.

## Data Availability

The datasets presented in this study can be found in online repositories. The names of the repository/repositories and accession number(s) can be found in the article/Supplementary Material.

## Abbreviations

HNSC, Head and Neck Squamous Cancer; TCGA, The Cancer Genome Atlas; WGCNA, Weighted Gene Co-expression Network Analysis; FPRS, Ferroptosis Prognosis-related Risk Score; CNV, Copy Number Variation; SNV, Simple Nucleotide Variation; RNA-seq, Transcriptome expression data; GEO, Gene Expression Omnibus database; OS, Overall Survival; ICB, Immune Checkpoint Blockade; TAF, Tumor-associated Fibroblasts; MDSCs, Myeloid-derived Suppressor Cells; TAM, Tumor-associated Macrophages; GSEA, Gene Set Enrichment Analysis.
