# Supplementary figures and images for: Identification and validation of a prognostic model based on ferroptosis-associated genes in head and neck squamous cancer
Source: Front Genet. 2022 Dec 1;13:1065546. doi: 10.3389/fgene.2022.1065546 (PMC9751480; doi:10.3389/fgene.2022.1065546)

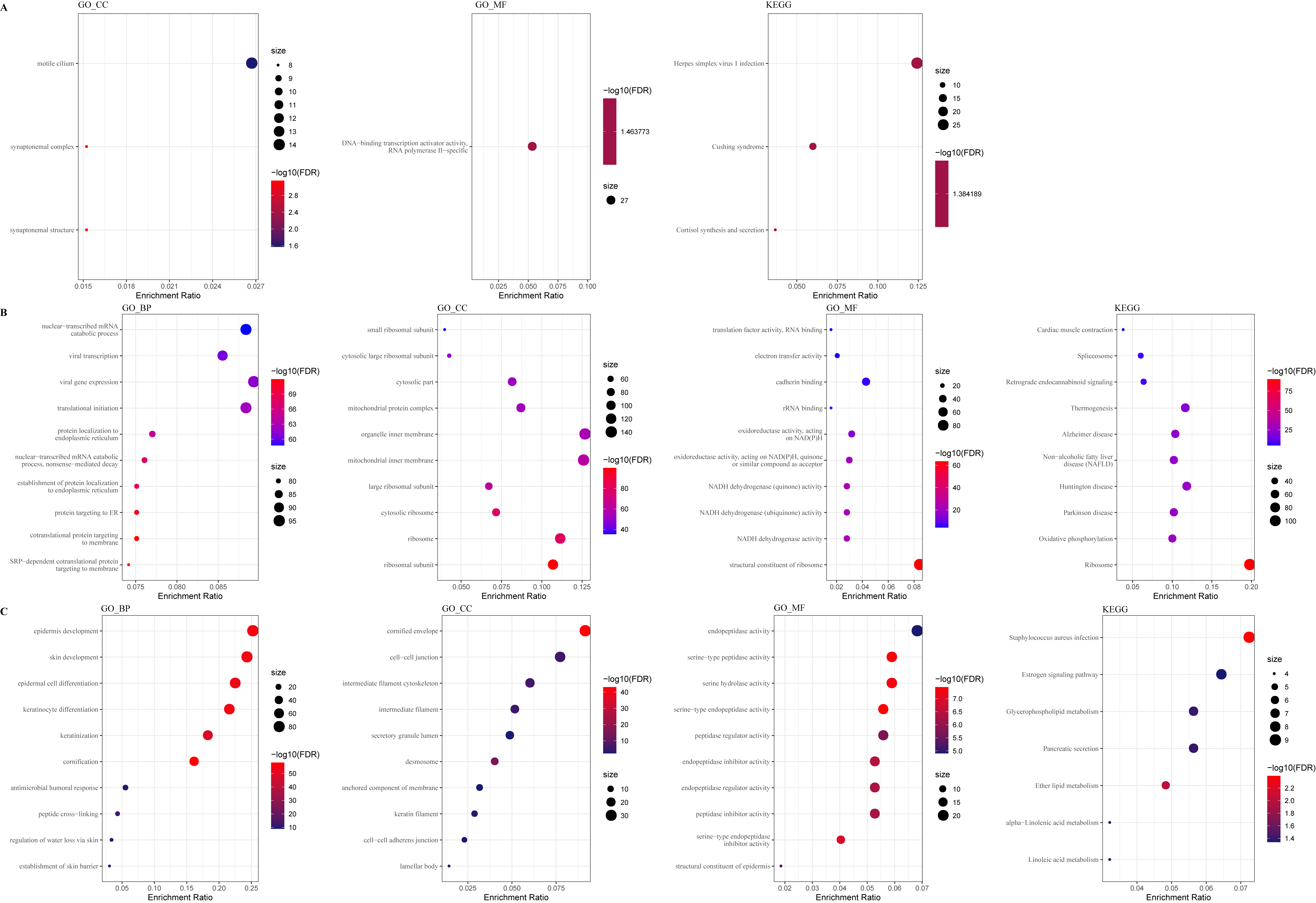

Supplement: Supplementary file 2 [file Image1.TIF]
